# The Various Forms of Nephrotic Syndrome in a Patient with Systemic Lupus Erythematosus

**DOI:** 10.1155/2020/7869216

**Published:** 2020-02-13

**Authors:** Sophia Lionaki, George Liapis, Kalliope Vallianou, Chrysovalantis Vergadis, Ioannis Boletis

**Affiliations:** ^1^Nephrology Department and Transplantation Unit, Laiko Hospital, National & Kapodistrian University of Athens, Faculty of Medicine, Athens, Greece; ^2^Pathology Department, Laiko Hospital, Athens, Greece; ^3^Radiology Department, Laiko Hospital, Athens, Greece

## Abstract

Kidney involvement is frequent in patients with systemic lupus erythematosus (SLE), although it may not be present from disease onset. Renal lupus itself is highly heterogenous with respect to the combination and/or severity of clinical and/or laboratory manifestations. This is a case of a 45-year-old Caucasian female with an established diagnosis of SLE, who presented four times with new onset of proteinuria during a follow-up time of ten years, since the diagnosis of SLE. Specifically, she experienced two episodes of lupus membranous nephropathy, and after she achieved remission, she developed twice overt nephrotic syndrome associated with new and biopsy proven lupus podocytopathy. All these episodes of nephrotic syndrome were combined with systemic symptoms, attributed to lupus itself, while serological activity of lupus was also noted. This case highlights the importance of performing a kidney biopsy in all patients with SLE who have new renal manifestations, including nephrotic proteinuria.

## 1. Introduction

Kidney involvement is very frequent in patients with systemic lupus erythematosus (SLE), although it may not be present from disease onset. Renal lupus itself is highly heterogenous with respect to the combination and/or severity of clinical and laboratory manifestations. The performance of each patient with kidney abnormalities is also variable but most importantly, different clinical settings and histopathological patterns may be found in the same patient over time. Nephrotic syndrome in patients with SLE is classically associated with proliferative glomerulonephritis or membranous nephropathy (WHO class III, IV, and V) [1, 2]. However, lupus podocytopathy associated with nephrotic proteinuria has been repeatedly reported in these patients [3], which is characterized by absent or scarce capillary wall immune complex deposition, or cellular proliferation, but diffuse effacement of the epithelial cell foot processes, identical to the histopathological pattern of minimal-change disease [4]. The diagnostic and classification criteria for lupus podocytopathy, which have been proposed by the American College of Rheumatology include the clinical diagnosis of full nephrotic syndrome combined with histopathology showing normal glomeruli by light microscopy, i.e., minimal-change disease (MCD), or focal segmental glomerulosclerosis (FSGS) with or without mesangial proliferation but with no endocapillary proliferation, necrosis, and/or crescents by immunofluorescence microscopy without deposits confined to the mesangium, and by electron microscopy diffuse and severe (typically >70%) foot process effacement [1]. It is considered to be rather pertinent than a simple coincidence [3], given the statistical unlikelihood of concurrent active lupus and idiopathic minimal change glomerulopathy. Accumulated evidence from clinical experience and epidemiologic observations indicate that lupus podocytopathy is related to the disease itself and is not random. Herein, we describe a case of a lupus patient, who developed overt nephrotic syndrome associated with new and biopsy proven lupus podocytopathy, following therapy and remission of previously diagnosed lupus membranous nephropathy. Furthermore, mesangial proliferation is the most common presentation (56%) followed by MCD (26%) and FSGS (18%). Undoubtedly, patients with FSGS have worse outcomes, higher rates of hypertension, and acute kidney injury on clinical presentation, with more severe tubulointerstitial involvement on kidney biopsy compared to patients with MCD and mesangial proliferative lesions.

## 2. Case Description

This is a case of a 45-year-old Caucasian female with an established diagnosis of SLE, who presented four times with new onset of proteinuria during a follow-up time of ten years since the diagnosis of SLE. The first time, 2 years after the diagnosis of lupus, she presented with proteinuria (mean value 3.3 grams), glomerular hematuria, and normal serum creatinine, combined with musculoskeletal symptoms, leukopenia, rash, positive antinuclear antibodies (ANA) >1 : 640, and low serum complements. The kidney biopsy clearly revealed lupus membranous nephropathy (WHO class V), i.e., subepithelial deposits and a full house pattern by immunofluorescence. She was started on therapy with oral methylprednisolone, cyclosporine, and hydroxychloroquine with complete remission of proteinuria and hematuria within 4 months (Table 1). However, 11 months later, she relapsed on therapy with new onset of proteinuria (14 grams per day), inactive urine sediment, normal renal function, fatigue, lymphadenopathy, generalized skin rash, and increased anti-dsDNA antibodies. A new kidney biopsy was performed, showing again lupus membranous nephropathy, with numerous, well-defined subepithelial, dense deposits by electron microscopy (EM). Mycophenolate mofetil was initiated in combination with glucocorticoids, and cyclosporine was withdrawn. She achieved complete remission again within a few months. Two years later, while on mycophenolate mofetil and low-dose prednisolone, she presented with new onset of nephrotic syndrome, i.e., edema anasarca and proteinuria (3.6 grams per day), combined with microscopic hematuria, skin rash, anemia, arthralgias, myalgias, high titers of antinuclear and anti-dsDNA antibodies, and low serum complement levels. The third kidney biopsy showed glomeruli of normal size, mild hyper cellularity, and normal thickness of the basement membrane. Immunofluorescence was negative, and EM revealed diffuse effacement of the podocyte foot processes >70%, consistent with lupus podocytopathy with the morphologic type of MCD and mild mesangial hyper cellularity. Mycophenolate mofetil was discontinued, and the patient was treated with high-dose glucocorticoids, i.e., 1 mg/kg body weight (BW) prednisolone daily, leading to remission within two months. However, tapering of glucocorticoids resulted in proteinuria increase, and thus, oral cyclophosphamide (2 mg/kg BW) was initiated for a total of three months with achievement of complete remission. The patient was maintained on 4 mg of methylprednisolone but unfortunately, six months later, she experienced again full-blown nephrotic syndrome. Of note, arterial pressure was well controlled with low dose of fosinopril. At this point, we performed a fourth biopsy, which was diagnostic again for lupus podocytopathy. She was treated with cyclosporine (3 mg/kg BW) and glucocorticoids (0.25 mg/kg BW) with a remarkable improvement of 24-hour urinary protein excretion, which returned to normal values within two months. Glucocorticoids were gradually reduced to 4 mg per day, while cyclosporine was tapered slowly until discontinuation. To date, she remains on hydroxychloroquine, with normal renal function and 24-hour urinary protein excretion, without extrarenal manifestations.

## 3. Discussion

Nephrotic syndrome or nephrotic range proteinuria in the context of SLE is often associated with identification of immune deposits in the glomerular capillary wall, with or without endocapillary proliferation and/or necrosis. However, there are reports of lupus patients who developed nephrotic syndrome in the absence of capillary wall immune deposits or cellular proliferation [3–10]. The lupus patient, who is described here, presented with nephrotic proteinuria with or without symptoms of nephrotic syndrome in four separate circumstances. In two occasions, histopathology revealed lupus membranous nephropathy (WHO class V) [1, 2] and in the other two, lupus podocytopathy, manifested as minimal-change disease. The probability of lupus podocytopathy, manifested as idiopathic minimal-change disease or idiopathic focal segmental glomerulosclerosis, has been estimated by Hertig et al. to be in <1 in 10,000 [3]. Yet, the observed prevalence of lupus podocytopathy was far higher than expected by chance, i.e., 2 per 132 patients with lupus involvement, in that study. Kraft et al. [4] reported 8 cases, derived from a pool of 470 renal biopsies performed in patients with SLE and abnormal renal indexes. Another series of seven lupus patients with diffuse podocyte effacement on renal biopsy manifested with nephrotic syndrome was presented by Dube et al. [5].

In our patient, like in most other cases, onset of nephrotic syndrome was observed in combination with clinical systemic symptoms of SLE and/or serological findings indicative of active disease and the discovery of lupus podocytopathy by histopathology. This is supportive of the notion that lupus podocytopathy is the result of active disease, more likely than the coexistence of two separate clinical entities who run concurrently [4]. More recently, Hu et al. studied fifty patients with lupus podocytopathy, counting for the 1.3% of all biopsies performed in lupus patients in their registry during the period 2000–2013 [11]. All had nephrotic syndrome and more than 70% foot process effacement [11]. Besides, a pathological transition can occur after renal relapse. In the study of Hu et al. [11], six patients changed from lupus podocytopathy to lupus nephritis class IV (*n* = 3) and class V (*n* = 3) while in our case transition occurred form class V to lupus podocytopathy. Based on their findings, Hu et al. proposed certain criteria for the diagnosis of lupus podocytopathy using clinical and pathological parameters. In this regard, and considering this entity as a distinct one, we need to establish a more specific connection between its pathogenesis and the activity of SLE [12] than the observation that these two occur concurrently. Furthermore, addition of this disease in the new revised classification of lupus nephritis is another requirement.

**Figure 1:**
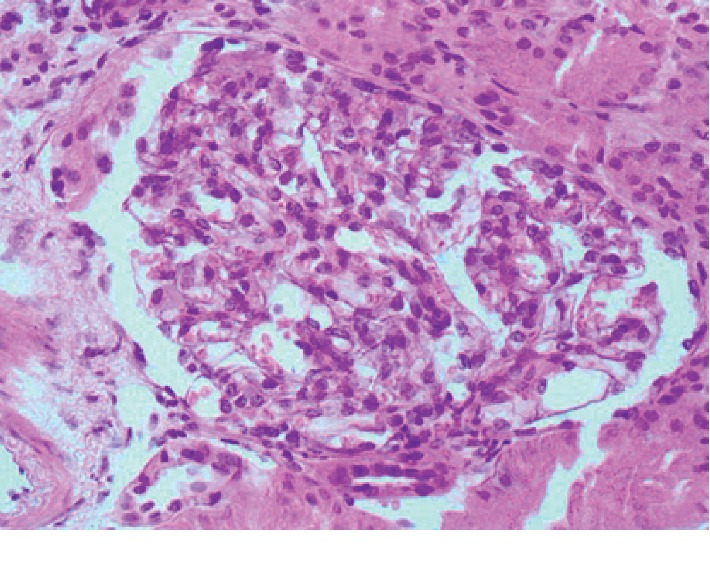
Glomerulus with no essential abnormalities; segmental and mild mesangial expansion at the time of lupus podocytopathy diagnosis (H&E, 400X).

**Figure 2:**
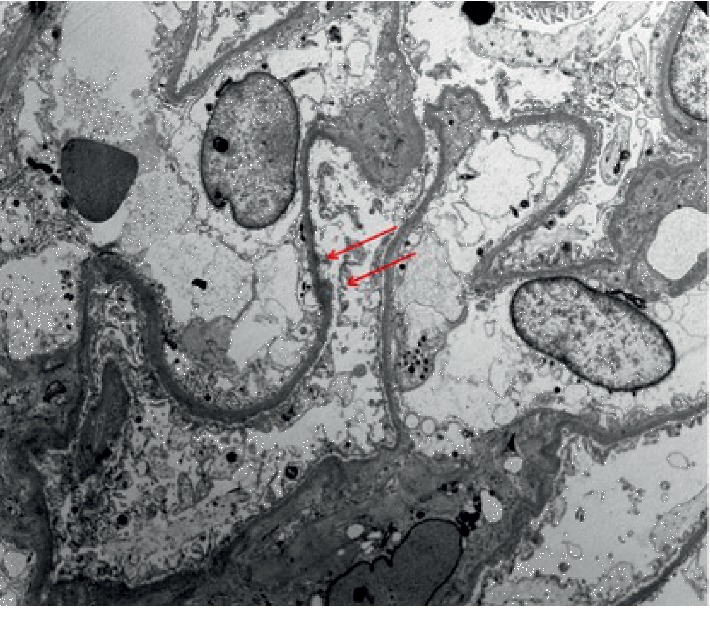
Diffuse foot process effacement (>70%) with scarce subepithelial deposits (red arrows), which represent residual scar from previous episodes of membranous nephropathy (uranyl acetate and lead citrate, 2800X).

The patient received therapy guided by the histopathological findings in each of these instances and finally achieved sustained remission. The treatment of lupus podocytopathy is based on observational and retrospective studies as the disease is quite rare. It generally follows the concept of therapy of MCD (glucocorticoids alone) or FSGS (combined with another immunosuppressive agent, i.e., calcineurin inhibitors, cyclophosphamide, or rituximab) considering that FSGS has a low rate of complete remission (22%) and a higher relapse rate (30–60%). Most patients with FSGS have a partial remission (55%) or do not respond to therapy (22%), with a longer median time to achieve remission (Figures 1 and 2).

Overall, the proposed criteria for the diagnosis of lupus podocytopathy in Oliva-Damaso and Chen [1, 13] are similar. In addition, Oliva-Damaso and co-authors proposed criteria for the clinical classification and also the inclusion of lupus podocytopathy in the classification of lupus nephritis, in between class II and Class III lupus nephritis. These authors proposed to include lupus podocytopathy in the International Society of Nephrology/Renal Pathology Society classification of lupus nephritis, as it is becoming clear now, that lupus podocytopathy is not a coexisting histological lesion but an independent type of renal involvement in SLE.

## 4. Conclusion

Lupus podocytopathy, which is characterized by diffuse foot process effacement without peripheral capillary wall immune deposits or glomerular proliferation, has been repeatably described in lupus patients with nephrotic syndrome and is becoming clear that it represents an independent type of renal involvement in patients with SLE. A kidney biopsy is mandatory in any new episode of renal involvement in lupus patients. Therapy should always be guided by histopathology, because renal indexes alone cannot precisely predict the type of the renal lesion.

## Figures and Tables

**Table 1:** Laboratory indexes and serological parameters at the time of kidney biopsy.

	24 h proteinuria (grams)	Glomerular hematuria	Serum creatinine (mg/dl)	Serum albumin (g/dl)	C3 (>80 mg/dl)	C4 (>10 mg/dl)	Antinuclear antibodies (<7 IU)
Biopsy #1	3.5	Yes	0.6	3.7	57	7	1/640
Biopsy #2	3.0	Yes	0.6	3.2	124	23	1/640
Biopsy #3	3.6	Yes	0.7	2.9	68	9	1/640
Biopsy #4	3.7	No	0.9	3.3	79	9.8	1/640
